# Anti-Inflammatory Mechanism of Polyunsaturated Fatty Acids in *Helicobacter pylori*-Infected Gastric Epithelial Cells

**DOI:** 10.1155/2014/128919

**Published:** 2014-06-02

**Authors:** Sun Eun Lee, Joo Weon Lim, Jung Mogg Kim, Hyeyoung Kim

**Affiliations:** ^1^Department of Food and Nutrition, Brain Korea 21 PLUS Project, College of Human Ecology, Yonsei University, Seoul 120-749, Republic of Korea; ^2^Department of Microbiology, Hanyang University College of Medicine, Seoul 133-791, Republic of Korea

## Abstract

*Helicobacter pylori* is an important risk factor for gastric inflammation, which is mediated by multiple signaling pathways. The aim of this study was to investigate the effects of polyunsaturated fatty acids (PUFAs), such as linoleic acid (LA), alpha-linolenic acid (ALA), and docosahexaenoic acid (DHA), on the expression of the proinflammatory chemokine interleukin-8 (IL-8) in *H. pylori*-infected gastric epithelial AGS cells. To investigate whether PUFAs modulate *H. pylori*-induced inflammatory signaling, we determined the activation of epidermal growth factor receptor (EGFR), protein kinase C-**δ** (PKC**δ**), mitogen-activated protein kinases (MAPKs), nuclear factor-kappa B (NF-**κ**B), and activator protein-1 (AP-1) as well as IL-8 expression in *H. pylori*-infected gastric epithelial cells that had been treated with or without PUFAs. We found that PUFAs inhibited IL-8 mRNA and protein expression in *H. pylori*-infected cells. **ω**-3 fatty acids (ALA, and DHA) suppressed the activation of EGFR, PKC**δ**, MAPK, NF-**κ**B, and AP-1 in these infected cells. LA did not prevent EGFR transactivation and exhibited a less potent inhibitory effect on IL-8 expression than did ALA and DHA. In conclusion, PUFAs may be beneficial for prevention of *H. pylori*-associated gastric inflammation by inhibiting proinflammatory IL-8 expression.

## 1. Introduction

*Helicobacter pylori (H. pylori)* is a Gram-negative bacterium that colonizes the human gastric mucosa [1].* H. pylori* infection is associated with various gastric diseases, such as chronic gastritis, peptic ulcer, and gastric adenocarcinoma [2]. The main pathogenic mechanism of* H. pylori*-induced inflammation is neutrophil infiltration into the epithelial cell layer [3]. This neutrophil activation can be indirectly mediated by chemokines, such as interleukin-8 (IL-8), which is secreted by gastric epithelial cells [4]. IL-8 is a potent mediator of the inflammatory response, by activating and recruiting neutrophils, basophils, and T cells to the site of infection [5]. A number of* in vivo* and* in vitro* studies have demonstrated that* H. pylori* infection induces IL-8 production in gastric cells and entails severe disease as documented by endoscopy and histology [6, 7].

*H. pylori* upregulates IL-8 production through multiple signaling pathways [8]. Nuclear factor-kappa B (NF-*κ*B) and activator protein-1 (AP-1) are essential transcription factors that regulate IL-8 expression [9, 10]. NF-*κ*B is an inducible transcription factor, composed of p50/p65 (heterodimer) or p50 (homodimer) [11]. It is retained in the cytoplasm by binding to its inhibitory protein, I*κ*B*α*. Extracellular stimuli trigger rapid degradation of I*κ*B*α* via the proteasome, allowing NF-*κ*B to translocate into the nucleus and bind to promoter regions of target genes [12]. AP-1 is a dimeric transcription factor composed of Jun and Fos subunits and binds to the AP-1 binding site [13]. It has been reported that transcription of IL-8 requires mainly NF-*κ*B activation, but both NF-*κ*B and AP-1 are indispensable for enhancing IL-8 mRNA transcription in* H. pylori*-infected gastric epithelial cells [14]. Activation of these transcription factors is mediated by mitogen-activated protein kinases (MAPKs) [15]. MAPKs are serine/threonine kinases that are activated in response to a variety of external signals. Three distinct pathways of MAPKs have been identified in mammalian cells: extracellular-regulated kinase (ERK), c-Jun N-terminal kinase (JNK), and p38 [16]. Previous studies have demonstrated that* H. pylori*-induced activation of MAPK mediates IL-8 expression by increasing NF-*κ*B and AP-1 DNA-binding activities [17, 18].

The mechanism by which* H. pylori* activates MAPKs has not been fully characterized. Previous studies have suggested a possible cascade of events: Ras-dependent activation of MAPKs via transactivation of receptor tyrosine kinases, such as epidermal growth factor receptor (EGFR), and Ras- and EGFR-independent activation of MAPKs via protein kinase C (PKC) [19]. EGFR is a transmembrane glycoprotein with intrinsic tyrosine kinase activity [20]. One of the important roles of EGFR activation is to transmit external signals into cells, which activates downstream signaling pathways, such as those involving MAPKs. A number of studies have demonstrated that* H. pylori* transactivates EGFR via activation and expression of the endogenous ligand heparin-binding EGF-like growth factor (HB-EGF) [21, 22] and subsequently stimulates ERK/JNK pathways [21, 23]. PKC is a family of protein-serine/threonine kinases that function as integrators of mitogenic signals in many cellular responses [24]. The role of PKC in* H. pylori* infection is not as clear as that of EGFR. However, a previous study demonstrated that PKC inhibitors significantly block* H. pylori* water extract-induced IL-8 production in MKN 45 cells [25]. Another study has shown that* H. pylori* infection activated PKC*δ* and subsequently the ERK pathway [26]. A recent study has demonstrated that a PKC inhibitor reduced AP-1 activation in* H. pylori*-infected gastric epithelial cells [27]. These results suggest that EGFR and PKC are potential mediators for regulating* H. pylori*-induced IL-8 expression.

Dietary fatty acids have been widely investigated for their influences on human health. Fatty acids are classified as saturated, monosaturated, and polyunsaturated fatty acids, based on their double-bond structure. Polyunsaturated fatty acids (PUFAs), which have multiple double bonds, have protective effects against inflammatory diseases, such as rheumatoid arthritis, inflammatory bowel disease, atherosclerosis, asthma, diabetes, and obesity [28–30]. Conjugated-linoleic acid and alpha-linolenic acid regulate inflammatory responses by downregulating proinflammatory eicosanoids [31, 32]. Recent studies have shown alternative anti-inflammatory actions of PUFAs, characterized by the modulation of expression of genes involved in inflammation in different cell lines [33–37]. Although epidemiological, clinical, and experimental studies have provided convincing evidence of anti-inflammatory effects of PUFAs, their roles and molecular mechanisms in* H. pylori* infection have not been explored fully.

To clarify the effects of PUFAs on* H. pylori*-induced gastric inflammation, we here investigated the anti-inflammatory effects of *ω*-6 fatty acid, linoleic acid (LA, C18:2), and *ω*-3 fatty acids: alpha-linolenic acid (ALA, C18:3), docosahexaenoic acid (DHA, C22:6), compared to those of a saturated fatty acid palmitic acid (PA, C16:0), on IL-8 expression in* H. pylori*-infected gastric epithelial AGS cells. Furthermore, we examined the effect of PUFAs on signaling pathways involved in IL-8 expression, to investigate the molecular mechanisms in which PUFAs are involved in* H. pylori*-infected gastric epithelial cells.

## 2. Materials and Methods

### 2.1. Reagents

PUFAs (PA, LA, ALA, and DHA) were purchased from Sigma-Aldrich (St. Louis, MO, USA). All fatty acids were dissolved in ethanol (final concentration: 10 mg/mL), aliquoted, sealed under N_2_ gas, and stored at −80°C. In each experiment, each fatty acid stock solution was freshly diluted with RPMI medium. AG 1478 (EGFR phosphorylation inhibitor, AG Scientific, San Diego, CA, USA), rottlerin (PKC*δ* inhibitor, Calbiochem, San Diego, CA, USA), U0126 (ERK inhibitor, Cell Signaling Technology, Danvers, MA, USA), and SP600125 (JNK inhibitor, Calbiochem) were dissolved in dimethyl sulfoxide at 10 mM in the stock solution. AG-1478 is a potent and specific inhibitor of EGFR tyrosine kinase with an IC_50_ of 3 nM [38]. Rottlerin is a specific inhibitor of PKC*δ* with an IC_50_ of 3–6 *μ*M [39]. U0126 is a specific inhibitor of MEK/ERK with an IC_50_ of 0.07 *μ*M for MEK 1 and 0.06 *μ*M for MEK 2 [40]. SP600125 is a potent, selective, and reversible inhibitor of three JNKs, with an IC50 of 40 nM for JNK1 and JNK2 and 90 nM for JNK3 [41].

### 2.2. Cell Culture

Human gastric epithelial cells (AGS) were obtained from the American Type Culture Collection (ATCC CRL 1739, Rockville, MD, USA) and grown in RPMI-1640 medium (GIBCO, Grand Island, NY, USA) supplemented with 10% heat-inactivated fetal bovine serum and antibiotic-antimycotic solution (100 U/mL penicillin and 100 *μ*g/mL streptomycin). The cells were cultured at 37°C in a humidified atmosphere of 95% air and 5% CO_2_. All experiments were carried out using an 80% confluent monolayer of these cells.

### 2.3. Bacterial Strain and* H. Pylori* Infection

An* H. pylori* strain (HP99) was isolated from the gastric mucosa obtained from a Korean patient with duodenal ulcer at Seoul National University [17]. HP99 was kindly provided by Dr. HC Jung (Seoul National University College of Medicine, Seoul, Korea). These bacteria were inoculated onto chocolate agar plates at 37°C under microaerophilic conditions using GasPak EZ Gas Generating Pouch Systems (BD Biosciences, San Jose, CA, USA). Prior to stimulation,* H. pylori* was harvested and then resuspended in antibiotic-free cell culture medium.* H. pylori* was added to the cultured cells at a bacterium : cell ratio of 500 : 1 in a 1-mL volume.

### 2.4. Fatty Acid Profile of AGS Cells

Lipid extracts were prepared from AGS cells and phospholipids were separated by thin layer chromatography [29]. The fatty acid composition of AGS cells was determined using gas chromatography (GC; Hewlett Packard 6890A GC, Miami, FL, USA), as described previously [30]. GC analysis was performed in triplicates.

### 2.5. Enzyme-Linked Immunosorbent Assay

AGS cells (1.5 × 10^5^ cells/mL) were seeded in 6-well plates. For time-course experiments, the cells were continuously cultured with* H. pylori* for various time periods (2, 4, 8, and 12 h). For fatty acid experiments, the cells were pretreated with PA, LA, ALA, or DHA (100 *μ*M) or ethanol vehicle for 24 h and cultured in the presence of* H. pylori* for another 4 h. Culture supernatants were centrifuged for 16,000 ×g (5 min at 4°C) and collected for assessing IL-8 levels in the medium using enzyme-linked immunosorbent assay (ELISA) kits (Biosource International, Inc., Camarillo, CA, USA).

### 2.6. Real-Time PCR (RT-PCR) Analysis of IL-8

IL-8 mRNA expression was analyzed by reverse transcription-polymerase chain reaction (RT-PCR) by coamplifying IL-8 with the housekeeping gene *β*-actin, which served as an internal control. The cells were cultured in the presence or absence of* H. pylori* for various time periods (0.5, 1, 1.5 2, and 3 h). For the fatty acid experiments, the cells were pretreated with PA, LA, ALA, DHA, or ethanol vehicle for 24 h and cultured in the presence of* H. pylori* for 2 h. The cells were isolated by Tri reagent (Molecular Research Center, Inc., Cincinnati, OH, USA). Total RNA was converted into cDNA by reverse transcription using a random hexamer and M-MLV reverse transcriptase (Promega Corp, Madison, WI, USA) at 23°C for 10 min, 37°C for 60 min, and 95°C for 5 min. cDNA was used for PCR with human-specific primers for IL-8 and *β*-actin. The sequences of the IL-8 primers were 5′-ATGACTTCCAAGCTGGCCGTGGCT-3′ (forward primer) and 5′-TCTCAGCCCTCTTCAAAAACTTCT-3′ (reverse primer), yielding a 297-bp PCR product. For *β*-actin, the forward primer was 5′-ACCAACTGGGACGACATGGAG-3′ and the reverse primer was 5′-GTGAGGATCTTCATGAGGTAGTC-3′, yielding a 349-bp PCR product. Fragments were PCR-amplified by 25–30 cycles, each consisting of denaturation at 95°C for 30 s, annealing at 60°C for 30 s, and extension at 72°C for 30 s. During the first cycle, the 95°C step was extended to 2 min, and on the final cycle the 72°C step was extended to 5 min. PCR products were separated on 1.5% agarose gels containing 0.5 g/mL ethidium bromide and were visualized by UV transillumination.

Real-time PCR was used to quantify IL-8 expression, using a Light Cycler (Roche, West Sussex, UK). cDNA was added in a SYBR Green Real-time PCR Master Mix (TOYOBO Co., Osaka, Japan) containing 10 pg/mL of forward and reverse primers for IL-8. For PCR amplification, the cDNA was amplified by 40 cycles, consisting of denaturation at 95°C for 15 s, annealing at 60°C for 15 s, and extension at 72°C for 30 s. During the first cycle, the 95°C step was extended to 30 s, and on the final cycle the 37°C step was extended to 1 min. *β*-actin was amplified in the same reaction, to serve as a reference gene.

### 2.7. Preparation of Nuclear Extracts

For time-response experiment, the AGS cells (2.5 × 10^6^ cells per 10 mL dish) were cultured with* H. pylori* for various time periods (0.5, 1, 2, and 4 h). For the fatty acid experiments, cells were pretreated with PA, LA, ALA, and DHA (100 *μ*M) for 24 h and further cultured with* H. pylori* for 1 h. The cells were harvested and washed with ice-cold phosphate-buffered saline (PBS) and extracted with lysis buffer containing 10 mM HEPES, 10 mM KCl, 1.5 mM MgCl_2_, 0.1% NP-40, 0.5 mM dithiothreitol (DTT), and 0.5 mM phenylmethylsulfonyl fluoride (PMSF). The nuclear pellets were resuspended in nuclear extraction buffer containing 10 mM HEPES, 420 mM NaCl, 0.2 mM EDTA, 1.5 mM MgCl_2_, 25% glycerol, 0.5 mM DTT, and 0.5 mM PMSF. Nuclear protein concentration was determined by Bradford assay, using the Bio-Rad protein assay solution (Bio-Rad Laboratories, Inc., Hercules, CA, USA).

### 2.8. Electrophoretic Mobility Shift Assay

NF-*κ*B gel-shift oligonucleotide (5′-AGTTGAGGGGACTTTCCCAGGC-3′) and AP-1 gel-shift oligonucleotide (5′-CGCTTGATAGTCAGCCGGAA-3′; Promega Corp, Madison, WI, USA) were labeled with [^32^P]-dATP (Amersham Bioscience, Piscataway, NJ, USA), using T4 polynucleotide kinase (Promega Corp). End-labeled probe was purified from unincorporated [^32^P]-dATP using a purification column (Amersham Biosciences, Buckinghamshire, UK) and recovered in Tris-EDTA buffer (TE). Nuclear extracts (5 *μ*g) were preincubated in buffer containing 12% glycerol, 12 mM HEPES, pH 7.9, 4 mM Tris-HCl, pH 7.9, 1 mM EDTA, 1 mM DTT, 25 mM KCl, 5 mM MgCl_2_, 0.04 *μ*g/mL Poly (dI-dC) · Poly (dI-dC) (Amersham Bioscience), 0.4 mM PMSF, and TE. The labeled probe was added and the samples were incubated for 30 min at room temperature. The samples were subjected to electrophoretic separation at 4°C on a nondenaturing 5% acrylamide gel. The gel was dried at 80°C for 40 min and exposed to a radiography film for 6–18 h at −80°C with intensifying screens.

### 2.9. Western Blotting for ERK, JNK, EGFR, and PKC*δ*

The cells (2.5 × 10^6^ cells per 10 mL dish) were pretreated with 100 *μ*M of PA, LA, ALA, or DHA, or ethanol vehicle for 24 h and cultured with* H. pylori* for various time periods. The cells were harvested and extracted with lysis buffer (10 mM Tris-HCl, pH7.4, 10% NP-40, and protease inhibitor cocktail). Cell extracts were subjected to 8–12% SDS-polyacrylamide gel electrophoresis and transferred to nitrocellulose membranes (Amersham Bioscience) by electroblotting. Membranes were blocked using 3–5% nonfat dry milk in TBS-T (Tris-buffered saline and 0.2% Tween 20) for 2 h at room temperature. The proteins were detected with antibodies against ERK, JNK, or phospho-JNK (Cell Signaling Technology, Beverly, MA, USA) and phospho-ERK, PKC*δ*, phospho-PKC*δ*, EGFR, and phospho-EGFR (Santa Cruz Biotechnology, Santa Cruz, CA, USA), diluted in TBS-T containing 3–5% dry milk, and incubated at 4°C for 16 h. After washing with TBS-T, primary antibodies were detected using horseradish peroxidase-conjugated secondary antibodies (anti-goat or anti-rabbit) and visualized using the ECL detection system (Santa Cruz Biotechnology), according to the manufacturer's instruction. Actin was used as a loading control.

### 2.10. Statistical Analysis

All values were expressed as mean ± S.E. of four different experiments. The statistical significance of differences was determined using Kruskal-Wallis analysis of variance. A value of *P* < 0.05 was considered to be statistically significant. The data were analyzed using SAS version 8 (SAS Institute, Inc., Cary, NC, USA).

## 3. Results

### 3.1. PUFAs Do Not Affect Cell Viability but Alter the Phospholipid Composition of AGS Cells

After culturing of AGS cells with PA, LA, ALA, and DHA (100 *μ*M) for 24 h, cell viability was not substantially different among treatment groups and the control (95–111%; data not shown). To examine whether AGS cells take up fatty acids and whether LA and ALA are converted to longer chain fatty acids, such as arachidonic acid, eicosapentaenoic acid, and DHA, the fatty acid composition of phospholipids in AGS cells was measured by GC, after treatment with individual fatty acid types for 24 h (Table 1). Each type of fatty acids used for treatment was incorporated into the phospholipids of AGS cells. LA and ALA were not converted to arachidonic acid, eicosapentaenoic acid, or DHA. Therefore, the effect of LA and ALA in the present study could be considered their sole effect, rather than the effects of their metabolites, on* H. pylori*-induced IL-8 expression in AGS cells.

### 3.2. PUFAs Inhibit IL-8 Expression in* H. Pylori*-Infected AGS Cells

**Figure 1:**
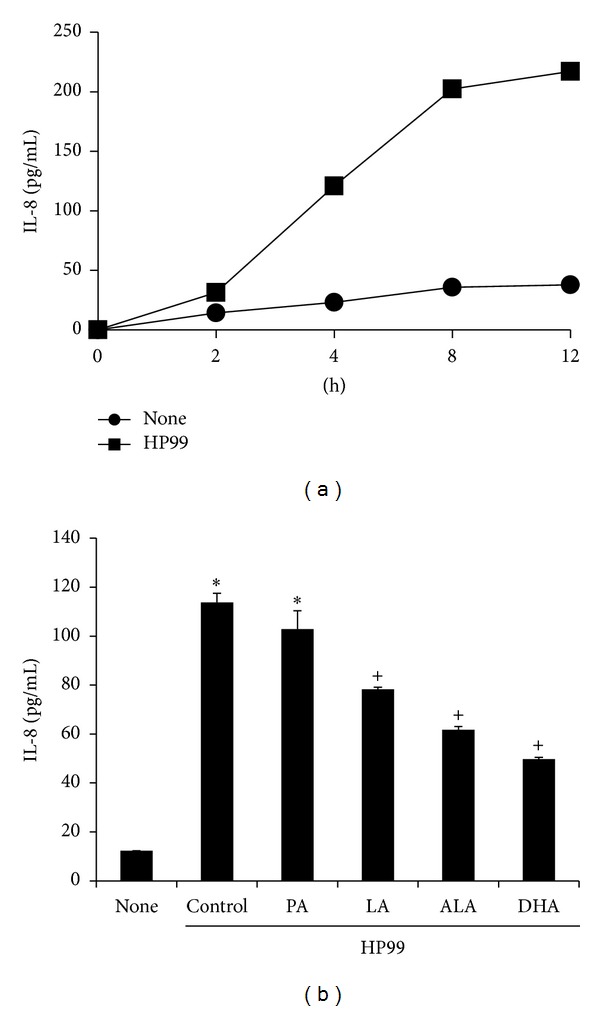
Time-dependent production of IL-8 and effects of PUFAs on* H. pylori*-infected AGS cells. (a) AGS cells were cultured with* H. pylori* for the indicated time periods (2, 4, 8, and 12 h) and IL-8 levels in the medium was measured by enzyme-linked immunosorbent assay. (b) AGS cells were pretreated with individual fatty acid (100 *μ*M) for 24 h and cultured with* H. pylori* for 4 h. Each bar represents mean ± S.E. of four separate experiments. *P < 0.01 versus None; ^+^ *P* < 0.01 versus control.

*H. pylori* increased IL-8 levels in the medium released from AGS cells in a time-dependent manner (Figure 1(a)). To investigate the effect of individual PUFAs on IL-8 secretion, AGS cells were pretreated with fatty acids for 24 h and then cultured with* H. pylori* for 4 h. Pretreatment with LA, ALA, and DHA inhibited the* H. pylori*-induced increase in IL-8 secretion (Figure 1(b)). Among PUFAs, DHA displayed the most favorable effect in reducing the increment in IL-8 levels in* H. pylori*-infected cells. PA did not decrease IL-8 secretion as much as did the other PUFAs.

### 3.3. PUFAs Inhibit IL-8 mRNA Expression in* H. Pylori*-Infected AGS Cells

**Figure 2:**
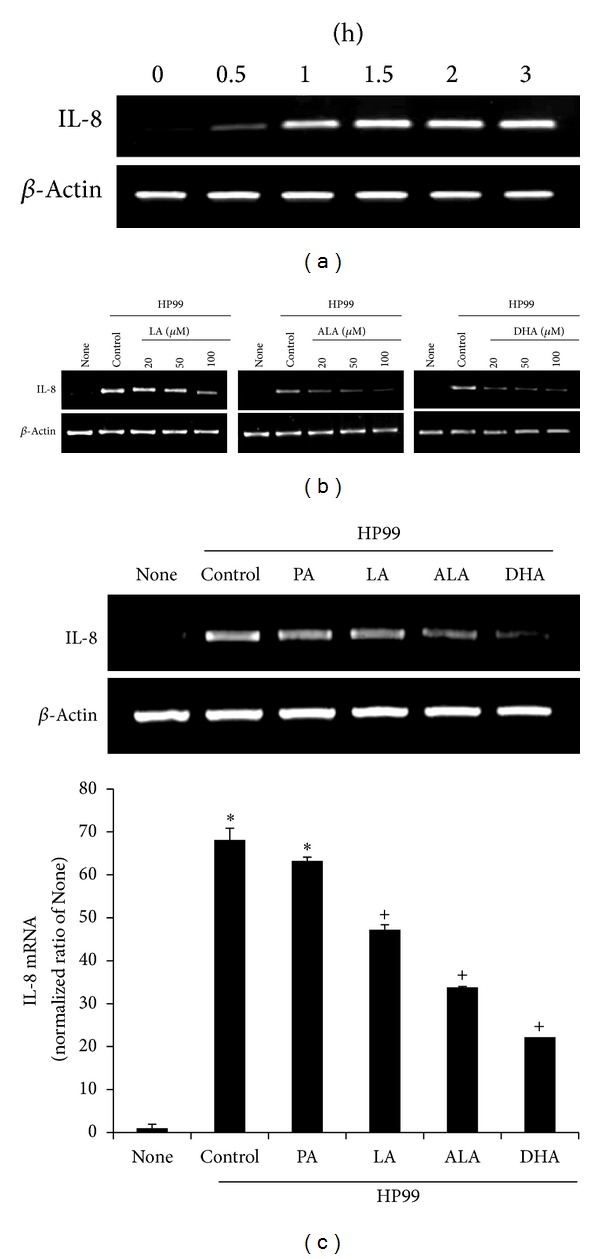
Effects of PUFAs on IL-8 mRNA expression in* H. pylori*-infected AGS cells. (a) AGS cells were cultured with* H. pylori* for the indicated time periods (0.5, 1, 1.5, 2, and 3 h) and IL-8 mRNA expression was measured by RT-PCR. (b) AGS cells were pretreated with various concentrations of LA, ALA, and DHA (20, 50, and 100 *μ*M) for 24 h and were then cultured with* H. pylori* for 2 h. (c) AGS cells were pretreated with individual fatty acid (100 *μ*M) for 24 h and then stimulated with* H. pylori* for 2 h. mRNA levels of IL-8 were quantified by real-time PCR. Data was normalized to *β*-actin level and expressed as relative ratio to None. Each bar represents mean ± S.E. of four separate experiments. *P < 0.01 versus None; ^+^ *P* < 0.01 versus control.

*H. pylori* induced mRNA expression of IL-8 time-dependently. IL-8 mRNA expression levels peaked at 1.5-h of culture and maintained at this level until 3-h of culture (Figure 2(a)). Various concentrations (20, 50, and 100 *μ*M) of PUFAs were used to pretreat the cells. After a 24-h treatment with PUFAs,* H. pylori* was added to the cells, and cells were cultured for another 2 h prior to monitoring IL-8 mRNA expression. LA, ALA, and DHA inhibited IL-8 mRNA expression in a dose-dependent manner (Figure 2(b)). In another experiment, AGS cells were pretreated with individual fatty acids (100 *μ*M) for 24 h and the expression of IL-8 mRNA was determined (Figure 2(c), upper panel) and quantified (Figure 2(c), lower panel) after culture with* H. pylori* for 2 h. Consistent with IL-8 protein expression, pretreatment of AGS cells with LA, ALA, and DHA decreased IL-8 mRNA expression, as compared to the cells only infected with* H. pylori*. PA did not inhibit* H. pylori*-induced IL-8 mRNA expression in AGS cells.

### 3.4. PUFAs Suppress* H. Pylori*-Induced Activation of NF-*κ*B and AP-1 in AGS Cells

**Figure 3:**
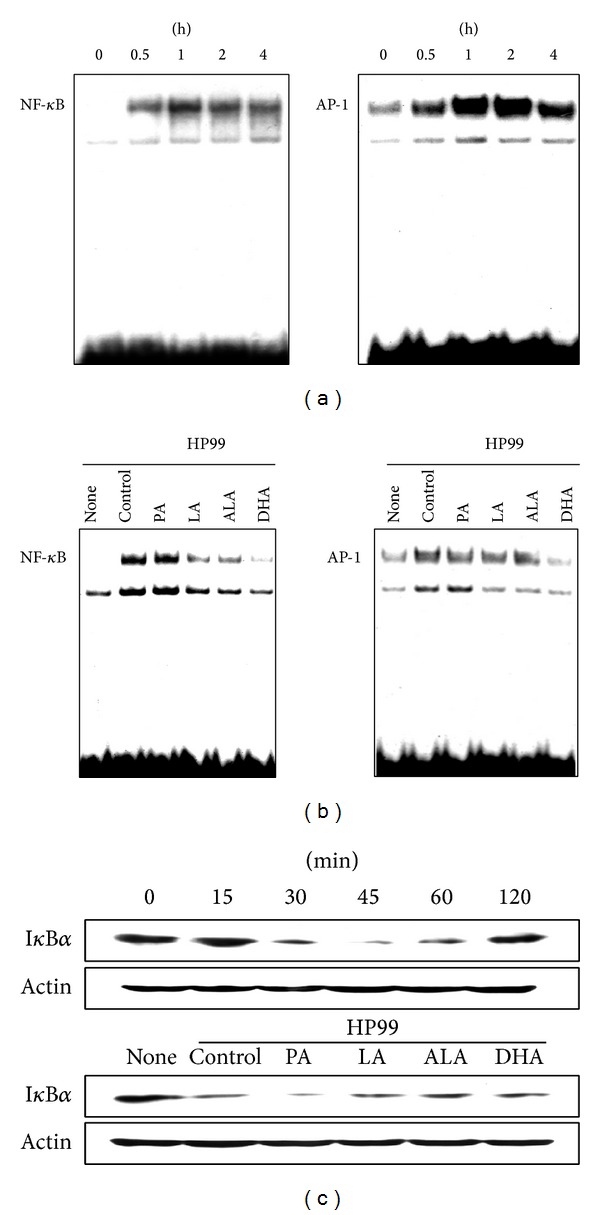
Effects of PUFAs on* H. pylori*-induced activation of NF-*κ*B and AP-1. (a) AGS cells were infected with* H. pylori* for indicated time periods (0.5, 1, 2, and 4 h). Nuclear extracts were harvested and the DNA-binding activities of NF-*κ*B- and AP-1 were determined by electrophoretic mobility shift assay. (b) AGS cells were pretreated with individual fatty acid (100 *μ*M) for 24 h and stimulated with* H. pylori* for 1 h. (c)* H. pylori*-induced degradation of I*κ*B*α* was analyzed by western blotting at the indicated time points (15, 30, 45, 60, and 120 min; upper panel). AGS cells were pretreated with individual fatty acid (100 *μ*M) for 24 h and infected with* H. pylori* for 30 min (lower panel). Actin was used as a loading control.

To determine whether PUFAs inhibit the activation of transcription factors involved in IL-8 expression, DNA-binding activities of NF-*κ*B- and AP-1 were assessed. Activations of NF-*κ*B and AP-1 were observed at 30 min of culture and peaked at 1 h (Figure 3(a)). Pretreatment of AGS cells with LA, ALA, and DHA suppressed* H. pylori*-induced activation of NF-*κ*B and AP-1, determined at 1 h (Figure 3(b)). In parallel with NF-*κ*B activation, degradation of I*κ*B*α* was shown at 30 min of culture, which was decreased by pretreatment of LA, ALA, and DHA in* H. pylori*-infected AGS cells (Figure 3(c)).

### 3.5. PUFAs Inhibit* H. Pylori*-Induced Activation of ERK and JNK in AGS Cells

**Figure 4:**
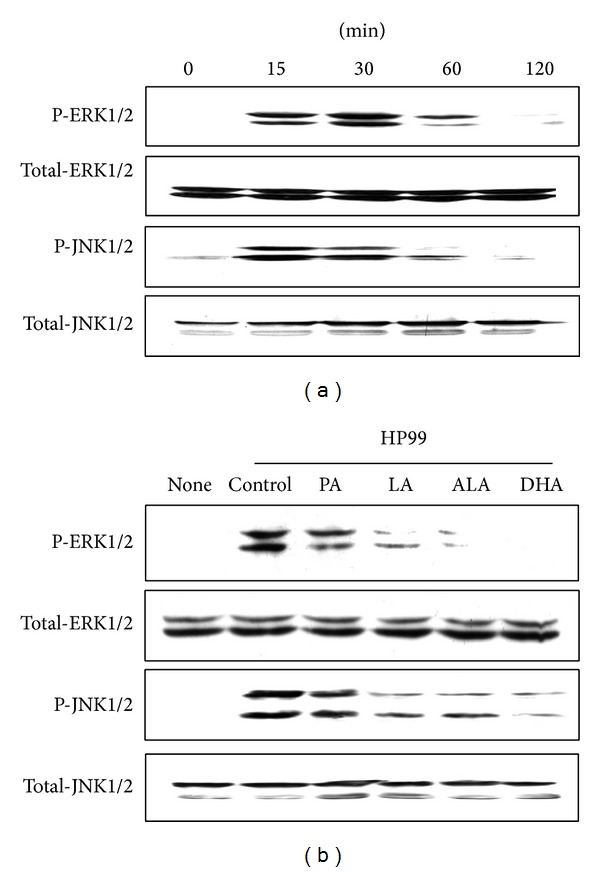
Effects of PUFAs on activation of ERK and JNK in* H. pylori*-infected AGS cells. (a) AGS cells were infected with* H. pylori* for the indicated time points (15, 30, 60, and 120 min). Phosphospecific and total forms of ERK and JNK were measured by western blot analysis. (b) AGS cells were pretreated with individual fatty acid (100 *μ*M) for 24 h and then cultured with* H. pylori* for 30 min.

To investigate the effects of PUFAs on* H. pylori*-induced activation of MAPKs, the phosphospecific forms of ERK and JNK were determined.* H. pylori* induced phosphorylation of ERK and JNK at 15 min, which continued up to 30 min of culture (Figure 4(a)). Pretreatment of AGS cells with LA, ALA, and DHA inhibited* H. pylori*-stimulated phosphorylation of ERK and JNK, as determined at 30 min (Figure 4(b)). Total forms of ERK and JNK were not changed by* H. pylori* infection and fatty acids treatment in AGS cells.

### 3.6. ALA and DHA Suppress* H. Pylori*-Induced Transactivation of EGFR, Which Modulates ERK and JNK Activation in AGS Cells

**Figure 5:**
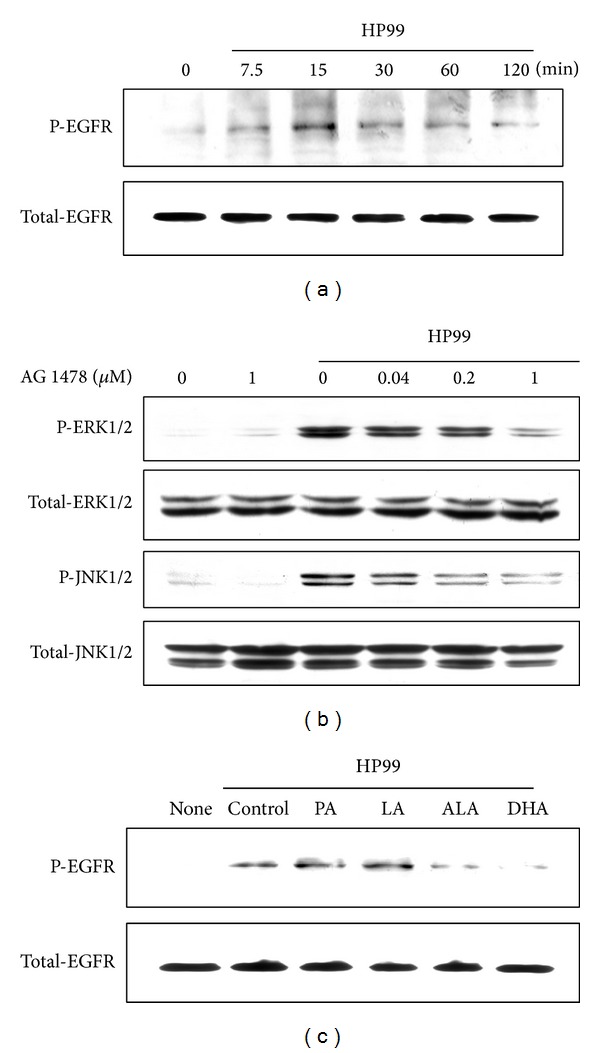
Effects of PUFAs on EGFR activation in* H. pylori*-infected AGS cells. (a) AGS cells were infected with* H. pylori* for the indicated time points (7.5, 15, 30, 60, and 120 min). Phosphospecific and total forms of EGFR were determined by western blotting. (b) AGS cells were pretreated with various concentrations of tyrphostin AG1478 (0.04, 0.2, and 1 *μ*M), a specific EGFR inhibitor, for 1 h prior to* H. pylori* stimulation The cells were then infected with* H. pylori* for 30 min and phospho-ERK and phospho-JNK levels were determined by western blotting. (c) AGS cells were pretreated with individual fatty acid (100 *μ*M) for 24 h and were then infected with* H. pylori* for 15 min.

To assess whether* H. pylori* infection activates EGFR in AGS cells, phosphorylation of EGFR was examined. EGFR phosphorylation was observed at 7.5 min and peaked at 15 min in* H. pylori*-infected cells; this level was maintained until 120 min of culture (Figure 5(a)). To examine whether* H. pylori*-induced activation of EGFR modulates ERK and JNK pathways, the cells were treated with tyrphostin AG1478, a specific EGFR tyrosine kinase inhibitor, for 1 h. The effect of AG1478 on* H. pylori*-induced activation of ERK and JNK was then determined. AG1478 attenuated* H. pylori*-induced activation of ERK and JNK at 30 min of culture in a dose-dependent manner (Figure 5(b)). In addition, pretreatment of cells with ALA and DHA for 24 h inhibited the phosphorylation of EGFR at 15 min of culture, while pretreatment with PA and LA did not reduce EGFR phosphorylation (Figure 5(c)).

### 3.7. PUFAs Inhibit* H. Pylori*-Induced Activation of PKC*δ*, Which Regulates ERK and JNK Activation in AGS Cells

**Figure 6:**
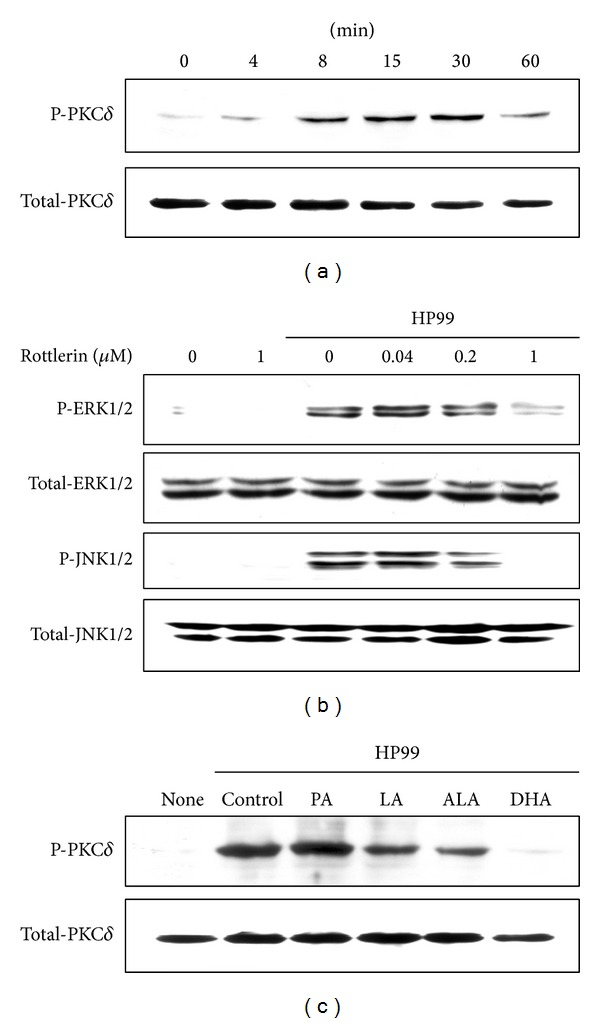
Effects of PUFAs on PKC*δ* activation in* H. pylori*-stimulated AGS cells. (a) AGS cells were infected with* H. pylori* for the indicated time points (4, 8, 15, 30, and 60 min). Phosphospecific and total forms of PKC*δ* were determined by western blotting. (b) AGS cells were pretreated with various concentrations of rottlerin (0.04, 0.2, and 1 *μ*M), a specific PKC*δ* inhibitor, for 1 h and then cultured with* H. pylori* for 30 min. Phosphospecific and total forms of ERK and JNK in whole cell extracts were determined by western blotting. (c) AGS cells were pretreated with individual fatty acid (100 *μ*M) for 24 h and were then cultured with* H. pylori* for 8 min.

To elucidate the involvement of PKC*δ* activation in* H. pylori*-infected AGS cells, the level of the phosphospecific form of PKC*δ* was measured. Phosphorylation of PKC*δ* was observed at 8 min after* H. pylori* infection, which continued until 60 min (Figure 6(a)). Pretreatment of cells with a selective PKC*δ* inhibitor, rottlerin, for 1 h inhibited* H. pylori*-induced phosphorylation of ERK and JNK at 30 min (Figure 6(b)). Pretreatment of cells with PUFAs (LA, ALA, and DHA) for 24 h suppressed phosphorylation of PKC*δ* in* H. pylori*-infected cells, determined at 8 min of culture (Figure 6(c)). DHA showed a potent inhibitory effect on* H. pylori*-induced activation of ERK and JNK in AGS cells, while PA did not affect* H. pylori*-induced phosphorylation of PKC*δ* in AGS cells.

### 3.8. The PKC*δ* Inhibitor Rottlerin Does Not Inhibit EGFR Transactivation in* H. Pylori*-Infected AGS Cells

**Figure 7:**
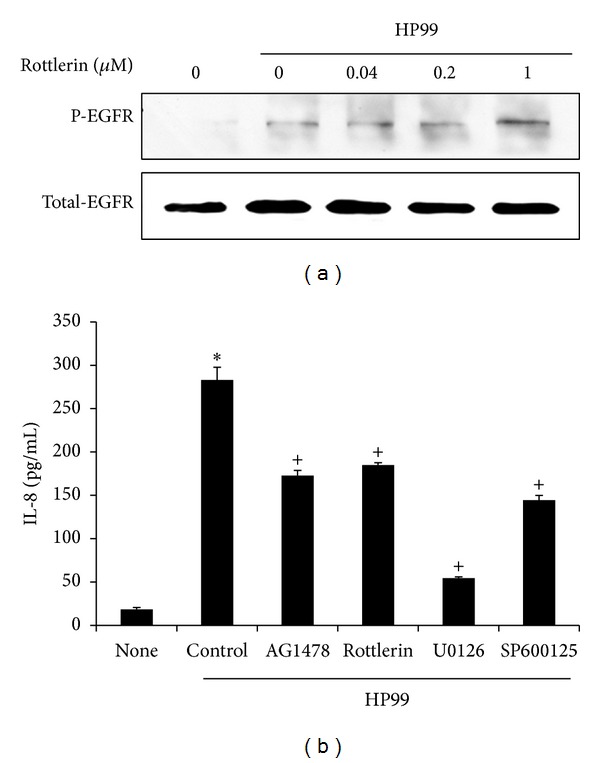
Effect of a PKC*δ* inhibitor on EGFR activation and effects of inhibitors of EGFR, PKC*δ*, ERK, and JNK on* H. pylori*-stimulated IL-8 production. (a) AGS cells were preincubated with various doses of rottlerin (0.04, 0.2, and 1 *μ*M) for 1 h and were then cultured with* H. pylori* for 15 min. Phosphospecific and total forms of PKC*δ* were determined by western blotting. (b) AGS cells were preincubated with AG1478 (1 *μ*M), rottlerin (1 *μ*M), U0126 (20 *μ*M), and SP600125 (20 *μ*M) for 1 h and were then infected with* H. pylori* for 4 h. IL-8 levels in the medium were measured by enzyme-linked immunosorbent assay. Each bar represents mean ± S.E. of four separate experiments. *P < 0.01 versus None; ^+^ *P* < 0.01 versus control.

To examine whether PKC*δ* modulates EGFR transactivation as an upstream signaling molecule,* H. pylori*-induced EGFR phosphorylation was assessed. AGS cells were pretreated with different concentrations of rottlerin for 1 h and cultured with* H. pylori* for 15 min (Figure 7(a)). Rottlerin did not inhibit phosphorylation of EGFR.

### 3.9. *H. Pylori*-Induced IL-8 Expression Is Inhibited by Suppression of EGFR, PKC*δ*, ERK, and JNK in AGS Cells

To investigate the involvement of EGFR, PKC*δ*, ERK, and JNK in* H. pylori*-stimulated IL-8 production, AGS cells were pretreated with AG1478 (EGFR inhibitor; 1 *μ*M), rottlerin (PKC*δ* inhibitor; 1 *μ*M), U0126 (ERK inhibitor; 20 *μ*M), and SP600125 (JNK inhibitor; 20 *μ*M) for 1 h and were then cultured with* H. pylori* for 4 h (Figure 7(b)). Inhibitors of EGFR, PKC*δ*, ERK, and JNK suppressed the* H. pylori*-induced increase in IL-8 levels in the medium. Among the inhibitors, U0126 exerted the most potent inhibitory effect on IL-8 production in* H. pylori*-infected cells.

## 4. Discussion

In the present study, we found that* H. pylori*, a Korean isolate (HP99), induced expression of the proinflammatory chemokine IL-8 via activation of EGFR and PKC*δ*, which then triggered downstream signaling characterized via the ERK/JNK pathways and activation of NF-*κ*B and AP-1 in gastric epithelial cells. Both *ω*-6 and *ω*-3 fatty acids inhibited PKC*δ* activation, while *ω*-3 fatty acids alone inhibited EGFR transactivation. These results suggest that PUFAs exert their anti-inflammatory effects by preventing upstream signaling that is stimulated by* H. pylori* infection.

An interesting finding of the present study is that individual fatty acid inhibited* H. pylori*-induced IL-8 expression to different extents. PA, a saturated fatty acid, did not inhibit* H. pylori*-induced IL-8 production and signaling cascades. This result is consistent with the studies showing that PA has little anti-inflammatory effect or even provokes proinflammatory cytokines in hepatocytes [42] and adipocytes [43]. Furthermore, *ω*-3 fatty acids exhibited more potent effects in downregulating IL-8 expression than did *ω*-6 fatty acid (LA). Among *ω*-3 fatty acids, DHA displayed a greater anti-inflammatory effect than did ALA. This result corresponds with previous studies showing that longer and more unsaturated fatty acids demonstrate more potent anti-inflammatory effects [44, 45].

There is a plausible explanation for the anti-inflammatory effects of *ω*-3 and *ω*-6 fatty acids. In the present study, the fatty acid composition of phospholipids derived from cells treated with different PUFAs suggests that LA and ALA are not converted into longer chain fatty acids, such as arachidonic acid and DHA. This may be because of the presence of too small amounts of desaturase and elongase in gastric epithelial cells. Therefore, the anti-inflammatory effects of LA and ALA are not attributable to their metabolites but to LA and ALA* per se*. The number of double bonds present in fatty acids is related to the magnitude of the anti-inflammatory effects of PUFAs. De Caterina et al. showed that the number of double bonds in long-chain fatty acids determines the extent of the inhibitory effects on endothelial activation by proinflammatory stimulation [45]. Recent studies suggested that different structures of fatty acids incorporated into phospholipid in cellular membrane confer different stability and fluidity to membranes and even affect membrane protein functions [46, 47]. A study has reported that *ω*-3 fatty acids, such as DHA and EPA, modified the lipid composition of membrane and altered EGFR and p38 signaling pathways in breast tumor cells [48].

In the present study, both an EGFR tyrosine kinase inhibitor and a PKC*δ* inhibitor suppressed* H. pylori*-induced activation of ERK/JNK and IL-8 expression in AGS cells, suggesting that EGFR and PKC*δ* are upstream mediators of* H. pylori*-induced IL-8 expression. It has been reported that an activated G-protein-coupled receptor (GPCR) leads to EGFR transactivation by activating PKC [49]. However, the results of the present study demonstrated that* H. pylori*-stimulated EGFR transactivation does not require PKC*δ* activation and that they may independently activate ERK and JNK pathways. It has been reported that MAPK can be directly activated by Raf phosphorylation, which is mediated by PKC*δ* activation in the absence of EGFR transactivation [19]. Our finding is also consistent with a previous study demonstrating that bradykinin, a blood vessel dilution agent, activated a dual signaling pathway involving PKC activation and EGFR transactivation in COS-7 cells [50, 51]. In regard to EGFR transactivation, GPCR is activated as an initial event of ligand binding to receptor and pro-HB-EGF is cleaved by an extracellular transmembrane metalloprotease to release mature HB-EGF for EGFR signaling [52]. Even though serial initial events including GPCR activation, cleavage of pro-HB-EGF, and HB-EGF release have not been determined in the present study, HB-EGF may mediate transactivation of EGFR in* H. pylori*-infected gastric epithelial cells. Taken together, PKC*δ* and EGFR activate ERK and JNK in* H. pylori*-infected cells. Our results indicated that *ω*-6 fatty acid has an inhibitory effect on PKC*δ* activation but not on EGFR transactivation, in* H. pylori*-infected cells. Therefore, PKC*δ* activation and EGFR transactivation may involve independent pathways.

A variety of foods and nutrients have been investigated as potential alternatives to antibiotics for* H. pylori* infection. Park et al. showed that red ginseng extract reduced* H. pylori*-induced cell damage and proinflammatory responses in human gastric epithelial cells [53]. Lee et al. demonstrated that capsaicin inhibited IL-8 production through suppression of NF-*κ*B DNA activity in gastric AGS and MKN45 cells [54]. Yang et al. showed that the combination of catechins and sialic acid reduced* H. pylori*-induced oxidative stress and apoptosis in human gastric cells and prevented gastritis in* H. pylori*-infected mice [55]. Wang and Huang showed that apigenin, a natural product of flavonoid, reduced expression of various genes involved in inflammation, through inhibition of NF-*κ*B DNA-binding activity and reactive oxygen species in MKN45 cells [56]. However, dietary intervention studies have been relatively less successful than* in vivo* and* in vitro* experimental studies. The effects of curcumin, vitamin C, vitamin E, selenium, and garlic have been investigated in individuals infected with* H. pylori* [57–60]. However, they showed limited or no effects on bacterial load or the inflammatory response induced by* H. pylori*. In the present study, *ω*-3 fatty acids exert anti-inflammatory effects by inhibiting PKC*δ* activation and EGFR transactivation, while *ω*-6 fatty acids inhibit PKC*δ* activation in* H. pylori*-infected AGS cells. PUFAs including *ω*-6 and *ω*-3 fatty acids showed inhibitory effects on* H. pylori* growth* in vitro* and* in vivo* studies, but no effect was found in a dietary intervention study [61–64]. Therefore, further carefully designed studies are needed to optimize the dose and duration of PUFAs treatments, considering the severity of* H. pylori* infection. In addition, more studies are required to examine not only the therapeutic but also the preventative effects of PUFAs on development of* H. pylori*-associated diseases.

## 5. Conclusion

We have demonstrated here the different anti-inflammatory effects of saturated, *ω*-6, and *ω*-3 fatty acids on* H. pylori* infection in human gastric epithelial cells. Furthermore, we showed that the molecular mechanism of the anti-inflammatory effects of PUFAs is associated with the modulated activation of membrane-related signaling proteins, such as EGFR and PKC*δ*, and their downstream signaling cascade including ERK/JNK and NF-*κ*B/AP-1. The present study suggests that PUFAs may prevent* H. pylori*-associated gastric inflammation. Further studies are required to evaluate the anti-inflammatory effects of PUFAs in* H. pylori* infection in* in vivo* animal experiments as well as in clinical studies.

## Figures and Tables

**Table 1:** Relative fatty acid composition of phospholipids in AGS cells.

(mol%)	None	HP				
		—	PA	LA	ALA	DHA
C14:0	3.45	3.23	2.92	3.13	2.60	3.53
**C16:0**	29.89	30.80	**42.18**	25.60	25.16	33.77
C16:1 (*ω*-7)	9.53	8.06	9.35	3.00	3.06	3.77
C18:0	12.78	13.93	10.68	14.24	14.85	16.13
C18:1 (*ω*-9)	23.50	23.48	16.02	11.42	13.75	14.71
C18:1 (*ω*-7)	4.47	4.49	3.22	3.04	2.99	3.62
**C18:2 (*ω*-6)**	3.51	3.52	3.30	**31.87**	2.34	2.44
**C18:3 (*ω*-3)**	0.33	0.24	0.17	0.15	**24.51**	0.24
C20:3 (*ω*-6)	1.69	1.62	1.78	0.53	1.08	0.83
C20:4 (*ω*-6)	8.06	7.69	7.63	4.50	6.87	4.96
C20:5 (*ω*-3)	0.20	0.35	0.29	0.62	0.36	1.49
C22:5 (*ω*-3)	1.24	1.26	1.21	0.98	1.20	0.73
**C22:6 (*ω*-3)**	1.35	1.34	1.24	0.91	1.23	**13.79**
